# Measurement of the Flow Field Generated by Multicopter Propellers †

**DOI:** 10.3390/s20195537

**Published:** 2020-09-27

**Authors:** Zbigniew Czyż, Paweł Karpiński, Wit Stryczniewicz

**Affiliations:** 1 Aeronautics Faculty, Military University of Aviation, 08-521 Dęblin, Poland; 2 Department of Thermodynamics, Fluid Mechanics and Aviation Propulsion Systems, Faculty of Mechanical Engineering, Lublin University of Technology, 20-618 Lublin, Poland; pawel.karpinski@pollub.edu.pl; 3 Aerodynamics Department, Łukasiewicz Institute of Aviation, 02-256 Warsaw, Poland; Wit.Stryczniewicz@ilot.lukasiewicz.gov.pl

**Keywords:** autogyro, flow velocity, gyrocopter, gyroplane, hybrid propulsion, multicopter, PIV, particle image velocimetry, wind tunnel

## Abstract

This paper presents the results of research on the airflow around a multirotor aircraft. The research consisted of the analysis of the velocity field using particle image velocimetry. Based on the tests carried out in a wind tunnel, the distribution of the velocity and its components in the vertical plane passing through the propeller axis were determined for several values of the angle of attack of the tested object for two values of airflow velocity inside the tunnel, i.e., *v_wt_* = 0 m/s and *v_wt_* = 12.5 m/s. Determining the velocity value as a function of the coordinates of the adopted reference system allowed for defining the range of impact of the horizontal propellers and the fuselage of the research object itself. The tests allowed for quantitative and qualitative analyses of the airflow through the horizontal rotor. Particular attention was paid to the impact of the airflow and the angle of attack on the obtained velocity field distributions.

## 1. Introduction

Several techniques for measuring and calculating aerodynamic properties and the phenomena occurring during airflow are used in aircraft aerodynamics studies. One of the trends involves the use of synergies of experimental research in wind tunnels and numerical calculations. In the case of numerical tests, computational fluid dynamics (CFD) is used at the design stage. Thus, it is possible to validate the assumptions of the conducted projects and avoid the costly preparation of models or prototypes for bench tests [1,2,3,4]. This study is an extended version of the paper entitled “Measurement of Air Flow Velocity around the Unmanned Rotorcraft,” presented during the 2020 IEEE International Workshop on Metrology for Aerospace in Pisa, Italy [5]. The numerical calculation stage is followed by experimental tunnel testing, in which visualization testing plays an important role. From the beginning of aeronautical research, visualization studies have been used to understand the phenomena occurring in all phases of aircraft flight. Currently, one of the most commonly used methods is particle image velocimetry (PIV), which is an optical method used for the qualitative visualization and quantitative measurement of flow velocity, e.g., the air around the examined object. The result of the measurement is a vector field of temporary velocity values. Based on this, it is possible to describe the phenomena occurring during the measurement. The flowing medium contains small particles (tracer particles) that act as markers [6]. While maintaining relatively small particle sizes in the main fluid, it is possible to accurately reflect the dynamics and flow path. The fluid with the suspended fine particles is illuminated such that the particles are visible in one selected plane. Particle image velocimetry involves photographing suspended particles placed in a flow and correlating the obtained images. Based on the movement of the tracer particles, a quantitative, two-dimensional flow image is obtained, which contains velocity vectors that specify the coordinate values and their direction.

**Figure 1:**
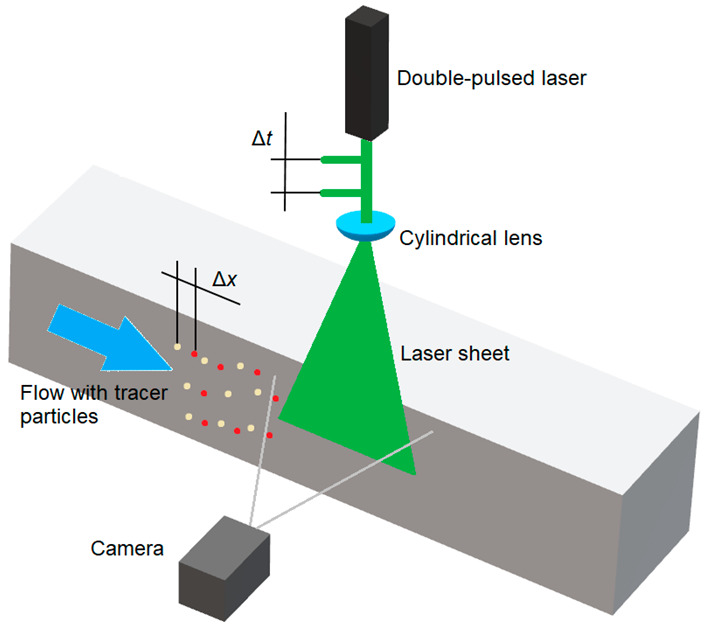
Diagram illustrating the principle of the particle image velocimetry (PIV) method.

Figure 1 shows a diagram explaining how the PIV technique works. A fluid with dispersed particles flows through the research domain. In the measurement space, there is a laser whose beam intersects the fluid in the selected plane. The laser light falls on particles suspended in the flowing medium such that they can be observed. A camera placed perpendicularly to the laser cutting plane is used to observe the particles in real time. In order to obtain the velocity field in the examined space, a simple physical relationship is used, i.e., *v* = d*x*/d*t*. The image obtained at time *t*_1_ is compared with the image at time *t*_2_ in terms of the temporary positions of the particles suspended in the medium. Knowing the time difference and distance difference for a given set of particles, it is possible to calculate the velocity at a given point of the measuring space at a given moment. For this purpose, post-processing is used, which involves performing a cross-correlation process. The presented analysis is carried out for the vertical and horizontal velocity components.

If there is a difference in the density of the medium and the particles sent into it, it is necessary to take into account the velocity lag in the constantly accelerating fluid. This situation occurs in the case of gases, where the density of particles is much higher than the density of fluid [7]. The velocity lag value can then be calculated using the formula:

(1) $$u_{s}=u_{p}-u=d_{p}^{2}\frac{\rho_{p}-\rho }{18\mu }a,$$

where:

*u_p_*—velocity of the tracer particles (m/s),
*u*—fluid velocity (m/s),
*ρ_p_*—density of the tracer particles (kg/m^3^).

In the PIV method, Brownian motion plays an important role, which involves the chaotic movements of particles in a fluid caused by the collisions of a suspension with fluid particles. The motion of a spherical particle, which the Stokes law can be applied to, is characterized by the diffusion coefficient introduced by Einstein [7]:

(2) $$D=\frac{KT_{a}}{3\pi \mu \cdot d_{p}},$$

where:

*d_p_*—particle diameter (m),
*K*—Boltzmann constant (J/K),
*T_a_*—absolute fluid temperature (K),
*μ*—dynamic viscosity of the fluid (Pa·s).

The PIV technique is currently widely used in aerodynamic research, both in the field of basic sciences and commercial research, for low and high flow rates. The application of PIV to helicopter rotor aerodynamic measurements was described in detail in Reference [8]. In Reference [9], on the basis of the conducted analyses, an image of the vortex ring on a helicopter’s rotor was obtained, and in Reference [10], a study of the temporal and spatial turbulence scales using a PIV system with a high measurement frequency was presented. A vortex ring was also examined using this method in Reference [11]. The rotor wake in ground effect and its investigation in a wind tunnel using the PIV technique was presented in Reference [12]. References [13,14] provide examples of the application of the PIV technique for testing transonic and supersonic flows, where the accurate representations of the shock wave position and the wave shape were of great importance. In Reference [15], calibration tests of the nozzle in the tunnel were performed with Mach numbers equal to 3.5 and 4.5. The measurements using modern PIV systems are characterized by an increasing frequency and a spatial resolution, thus making it possible to examine complex flow phenomena [16]. The development of PIV systems is a consequence of the progress in laser and computer technologies, which facilitate the implementation of advanced image processing algorithms [7,17,18]. In this work, the PIV technique was used to determine the aerodynamic interference of rotor streams of an innovative multirotor aircraft. The results obtained can be used to optimize the new design.

The PIV method can be used to analyze interference or aerodynamic interaction of objects. An example is Reference [19], in which the authors examined the interaction between a helicopter rotor and a ship hull on its board during the landing process.

The PIV technique has been constantly improving, as confirmed by several scientific papers devoted to this topic. References [20,21] present a new system for large-scale tomographic PIV in low-speed wind tunnels. For tracer particles, sub-millimeter helium-filled soap bubbles were used. It was proved that using this solution significantly improved the light scattering compared to micron-sized droplets. The authors of Reference [22] used a pulse-burst laser as part of a time-resolved PIV method. This made it possible to visualize the flow under high-speed and turbulence conditions.

PIV tests can be combined with simulation tests performed using the CFD method. An example is Reference [23], in which a vertical axis wind turbine of the Savonius type was studied. The simulation tests were validated through wind tunnel tests using the PIV method. A similar approach was used in Reference [24], in which a cross-ventilation flow for different isolated building configurations using CFD and PIV methods was studied. This method is often used with other measuring methods, e.g., embedded laser doppler velocimetry (ELDV). This approach was used by the authors of Reference [25], where the boundary layer profile and the characteristics of the flow velocity distribution, close to the leading edge of a helicopter blade profile, were examined using both techniques. The authors of Reference [26] used the same methodology in order to examine vortex shedding and the wake–wake interaction in a transonic turbine stage.

A comprehensive analysis of the application of the PIV method for studying the aerodynamic loads of airfoils and aircraft propellers is presented in Reference [27]. The authors focused on the method of determining the pressure field using the flow velocity field obtained by the PIV method, among other aspects. The research in this area is also presented in Reference [28]. Furthermore, Reference [29] presented various ways of using the PIV technique for the rotor aerodynamics analysis.

The PIV method is used to study the distribution of flow velocities outside and inside an aircraft. In Reference [30], the authors tested tactical jet aircraft nozzles, while in Reference [31], they analyzed the air distribution inside the passenger cabin. In Reference [32], the distribution of the flow velocity around the helicopter structure was examined in 37 different regions for two model configurations (isolated fuselage and fully-equipped model) in four different flight conditions. These studies are more widely presented in Reference [33]. Interesting research on helicopter aerodynamics using the PIV method is presented in Reference [34]. The wake of a full-scale UH-60A rotor was tested in forward flight in a wind tunnel.

Flow field tests are extremely important from the point of view of analyzing the phenomenon of aerodynamic interference. Research on this subject is carried out in the largest research and scientific centers in the world [35]. It should be noted that flow visualization obtained as a result of computer model solutions is one of the most important instruments for analyzing the solutions obtained, and is also a tool for the in-depth analysis of the impact of flow interference phenomena on the resultant loads of rotorcraft. The variability of the flow field in time, the interpenetration of vortex traces, and the pulsations of the disturbance field can be assessed both qualitatively and quantitatively by observing the image of this phenomenon on the computer screen, which involves introducing the selectivity of interference components where possible. Based on the tests carried out using PIV image anemometry, airflow velocity profiles were developed as a function of the distance from the propellers. On this basis, it is possible to determine the range of the additional multirotor drive system. The analysis of the velocity field around the tested object allows for the selection of the installation location (height) of both the rotor working in autorotation relative to the fuselage and other components to minimize the impact of the additional propulsion system.

## 2. Research Object and Methodology

### 2.1. Research Object

**Figure 2:**
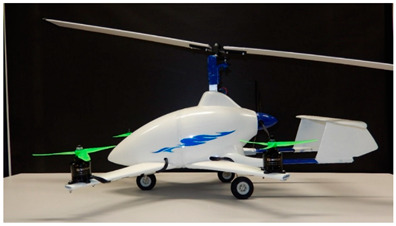
View of the model of the tested multirotor aircraft.

The research object was a model of an aircraft equipped with a hybrid propulsion system. The aircraft is an innovative structure combining the features of a multirotor and a gyroplane. The concept combined the advantages of a gyroplane (a light machine with a simple design and was cheaper to operate) with the advantages of a multirotor design that allowed for shortening the take-off distance or even vertical take-off and landing. The use of additional propulsion ensured flight stabilization at low speeds and increased the safety of the entire aircraft. The main rotor rotated, as in a classic gyroplane, due to the phenomenon of autorotation, while the set of additional propellers was driven by brushless electric motors. The combination of these two different propulsion systems resulted in an innovative means of individual air transport and a machine capable of performing various types of unmanned missions. The structure of the tested aircraft consisted of four horizontal propellers, a pushing propeller, and a two-blade main rotor. The basic technical parameters of the research object are presented in Table 1, while the design of the discussed concept is shown in Figure 2.

**Figure 3:**
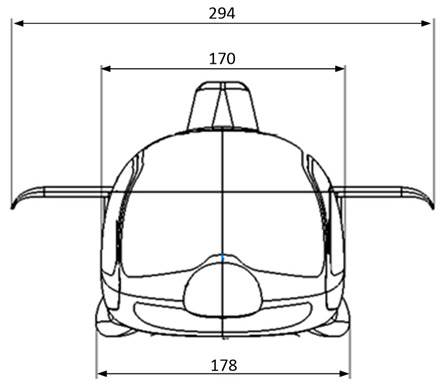
Geometric dimensions (mm) of the test object (front view).

**Figure 4:**
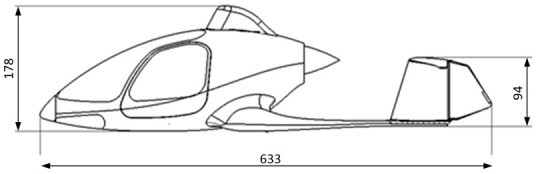
Taifun gyroplane-geometrical dimensions (mm) of the object (view from the left).

The geometric model of the aircraft was based on the structure of the Taifun gyroplane, which was designed by the company AVIATION ARTUR TRENDAK (Jaktorów-Kolonia, Poland). The gyroplane model was transferred for the purposes of ongoing works due to the cooperation between entities. Figure 3 and Figure 4 show the characteristic dimensions of the fuselage with the tail boom, which was used to develop the design of the hybrid aircraft. These are the real dimensions of the Typhoon gyroplane. The model used for the research was made on a scale of 1:8.

The drive unit of the examined object consisted of several cooperating key elements, i.e., propellers, engines, control regulators, power supply (battery), flight controller, and radio-controlled (R/C) devices. A set of propellers and engines were responsible for generating thrust. Blades with the NACA-9H-12 profile were used. Each set generated about 1800 g of thrust from each engine. This value was required to lift the aircraft, including sufficient reserves to perform maneuvers during the flight, based on the assumption that the main rotor did not generate lift. The additional drive units were equipped with T-MOTOR MT2814 770 KV engines. The design of the engines used in combination with 10-inch propellers allowed for generating the assumed thrust.

### 2.2. Methodology

**Figure 5:**
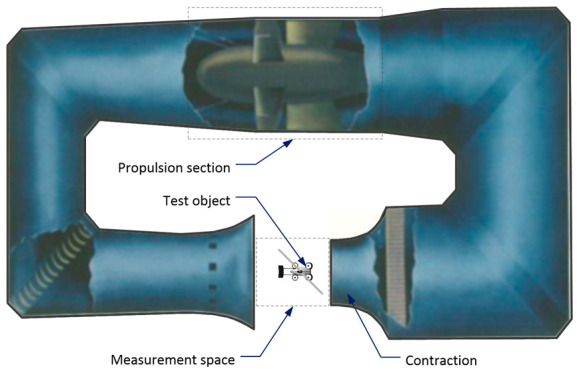
View of the used T-1 wind tunnel at the Polish Institute of Aviation [37].

The presence of additional propellers affects the airflow around the fuselage, which may result in the deterioration of the aerodynamic properties of the entire structure. Therefore, the research was aimed at analyzing the impact of additional horizontal propellers on the velocity field around the hybrid aircraft. The tests were carried out in a T-1 wind tunnel (Figure 5). This is a tunnel with a low-speed flow and continuous operation. The device has an open measuring space with a diameter of 1.5 m and a length of 2.0 m. The maximum air velocity in the tunnel equals 40 m/s, whereas the minimum is equal to 10 m/s. The airflow is generated by rotating adjustable fan blades driven by a 55 kW electric motor. During the measurements, the turbulence intensity was limited to 0.5% for the empty measuring space. The test stand is part of the tunnel measuring and control system, which is described in more detail in Reference [36].

**Figure 6:**
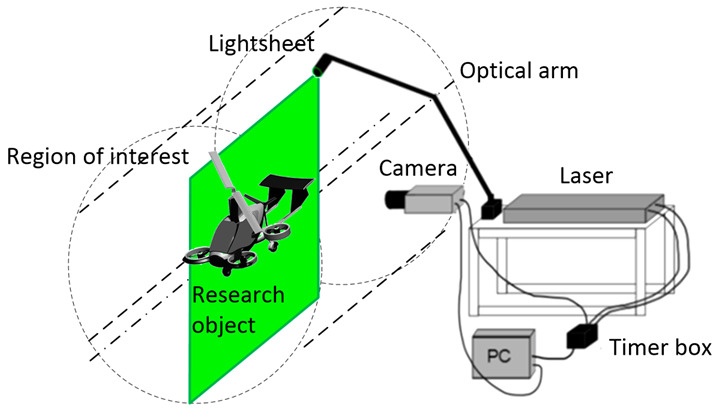
Diagram of the test stand for visualization of the flow around the research object (own study based on Reference [37]).

Figure 6 shows a test stand for visualizing the airflow around a multirotor aircraft. A detailed description of the measuring system is presented in References [9,38]. The velocity field visualization system was based on the PIV technique and consisted of two key components: a twin-head solid-state laser from Litron (Litron, Rugby, Warwickshire, England) (ND: YAG neodymium laser) and a PCO HiSense digital camera (PCO AG, Kelheim, Germany). The beam in each head was triggered using a given frequency and the time interval between the heads. Then, the beam was formed as a luminous cutting plane due to the cylindrical lenses. The laser light was introduced into the measuring chamber due to a special mounting arm. The tracer particles were carried out in the form of dispersed oil droplets generated on an air generator at a pressure of 1.5 bar. In this way, particles with an average size of 2 μm were obtained, which were then introduced into the tunnel channel through a nozzle. The Stokes criterion was verified. In the experiments, the DHES (dioctyl sebacate, di(2-ethylhexyl) sebacate) particles were used with a characteristic time of 2 μs. The selected particles followed the investigated flow based on their Stokes parameter value of less than 10^−1^ [7,39,40,41].

The aircraft model was placed in the tunnel in such a way that the axis of the tunnel coincided with the plane of symmetry of the fuselage of the research object. For the 0° angle of attack, the normal vector to the main rotor plane was perpendicular to the air velocity vector in the wind tunnel. The tests were carried out for several defined attack angle values. Samples were taken at various blade positions around the blade revolution. The inverted vertical and horizontal velocity values at the advanced blade might be a result of the experimental data corruption by light-sheet reflections.

**Figure 7:**
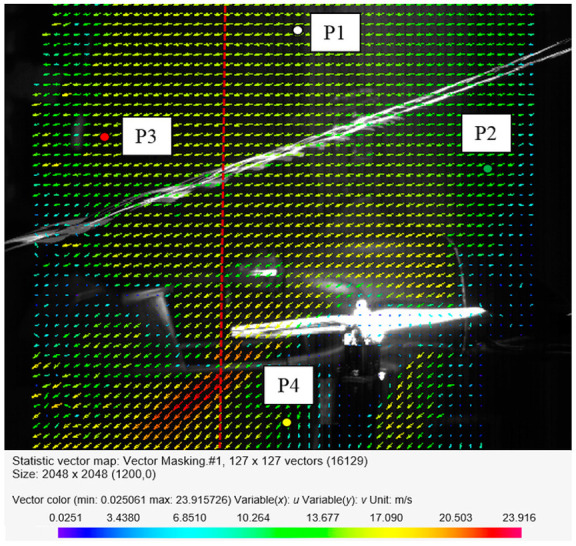
Location of the control points in the measurement space.

The camera was positioned in such a way that the observed area was 700 × 700 mm, which included the main rotor, the additional propeller at the rear of the body, the fuselage, and the stabilizers. It did not cover the whole frame of the camera due to the angle of propagation of the cutting plane being too small. The generation of the air velocity vectors using the PIV method was performed with a resolution of 5 mm. The universal outlier detection technique was used for PIV data validation [42]. The median test was modified by introducing a single threshold to the normalized vector residuals. The resulting algorithm is universal for a variety of different flow conditions and characteristics. In this study, the normalized residuals of the vectors were calculated from 5 × 5 mm adjacent vectors. For the selected interrogation window size of 32 × 32 px, the resulting vector velocity field had 16,129 vectors placed in a regular grid of 127 columns and 127 rows. The presentation of such a dense vector velocity field in this paper was difficult because the vectors have to be very small or they will overlap. For the clarity of the presentation, every second row and column of the vector field is presented. This allowed for presenting sufficiently large vectors for a clear presentation and to avoid overlapping. The presented velocity field was created by averaging an ensemble of 150 instantaneous velocity fields. The size of the ensemble was chosen based on the results of previous studies. The convergence of the control points located in four different locations was verified: P1—in the freestream above the model, P2—in front of the main rotor above the model fuselage, P3—behind the main rotor, and P4—in the small horizontal propeller wake. The location of the control points is presented in Figure 7. The red dashed line in Figure 7 corresponds to the RMS velocity profile for the selected *x*-coordinate.

**Figure 8:**
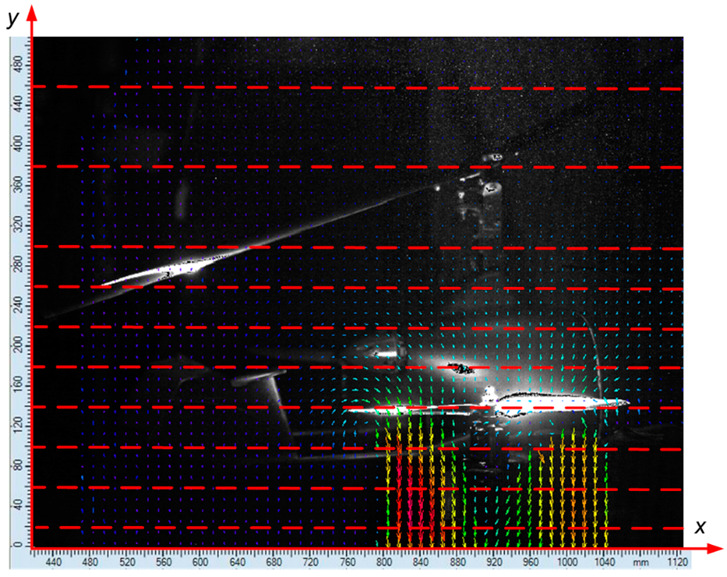
The adopted coordinate system of the examined area around the aircraft, along with the cross-sections considered (horizontal axis—*x*, vertical axis—*y*).

In order to present the flow field around the research object, the velocity distribution was analyzed for the selected values of the *y*-coordinate marked in red broken lines in Figure 8. The coordinate system for the measurement space was defined by two axes: *x* and *y*. The values of the *x*-coordinate increased toward the front of the aircraft. The *y*-axis was perpendicular to the *x*-axis. Due to the analysis of a fragment of the measurement area, the values on the *x*-axis did not start from zero.

Due to the fact that for a configuration of active propellers with a vertical thrust vector, the vertical airflow is forced, and thus the selected coordinates on the vertical *y*-axis were analyzed. Near the active propellers, the distance of the analyzed sections was 40 mm, while away from them, it was equal to 80 mm. The propeller axis of the additional drive unit passed through coordinate *x* = 880 mm. The propeller operating plane at *α* = 0° was at the height described with the coordinate *y* = 140 mm. The position of the main rotor head was described using coordinates *x* = 1000 mm and *y* = 380 mm, while the horizontal stabilizer was located at a height of approximately *y* = 200 mm (*α* = 0°).

**Figure 9:**
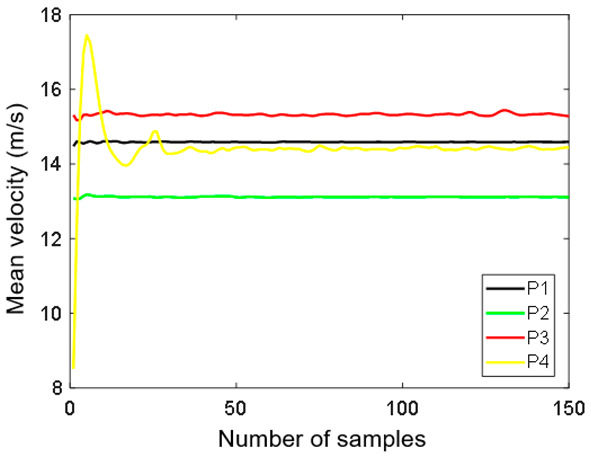
Convergence for increasing numbers of vector fields of the mean velocity at the control points.

**Figure 10:**
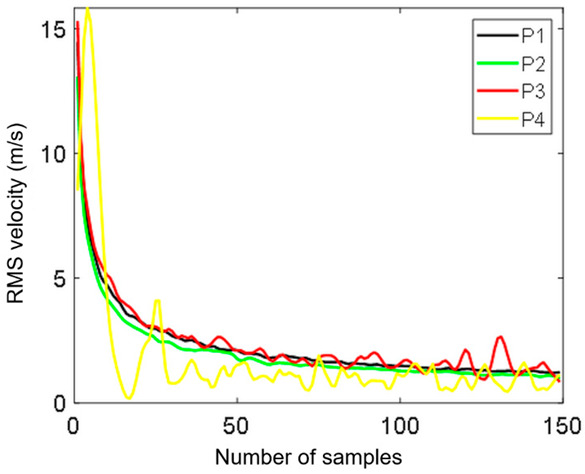
Convergence for the RMS velocity at the control points.

The number of samples used for averaging was sufficient to reach a steady value, as it can be seen in the convergence studies results presented in Figure 9 and Figure 10 [43,44]. Figure 9 shows the mean velocity as a function of the number of samples for the indicated control points P1–P4. The assumed number of samples was equal to 150, which allowed for obtaining convergence for the mean velocity in the control points. Moreover, the convergence of the RMS velocities at points P1–P4 was verified. The obtained results are presented in Figure 10. The RMS velocity magnitude *v*_RMS_ was calculated using:

(3) $$v_{\mathrm{RMS}}=\sqrt{v_{\mathrm{xRMS}}^{2}+v_{\mathrm{yRMS}}^{2}}$$

The uncertainty in the vectors of the instantaneous velocity field maps was estimated using the primary peak ratio technique [45]. The uncertainty of an individual velocity vector in the freestream did not exceed 0.5 m/s, while in the main rotor proximity, it was less than 1 m/s. An exceptionally high value of uncertainty of up to 2 m/s was estimated in the wake of the small horizontal propellers. This might have been caused by a loss of particle pairs due to the strong motion out of the plane. The uncertainty of the instantaneous velocity measurement at given *x*- and *y*-coordinates propagated to the uncertainty of the averaged results [43,44]. The standard deviation of the uncertainty velocity magnitude was equal to 0.1447 m/s at P1 and 0.2397 m/s at P4. According to the methodology given in Reference [46], the uncertainty of the mean velocity of 150 samples was equal to 0.0118 m/s at P1 and 0.0196 m/s at P4. The selection of points P1 and P4 for analyzing the uncertainty velocity was dictated by the fact that extreme flow conditions occurred at these points. At point P1 (the furthest from the test object), the lowest uncertainties were expected, and at point P4 (below the propeller), the highest uncertainties were expected.

**Figure 11:**
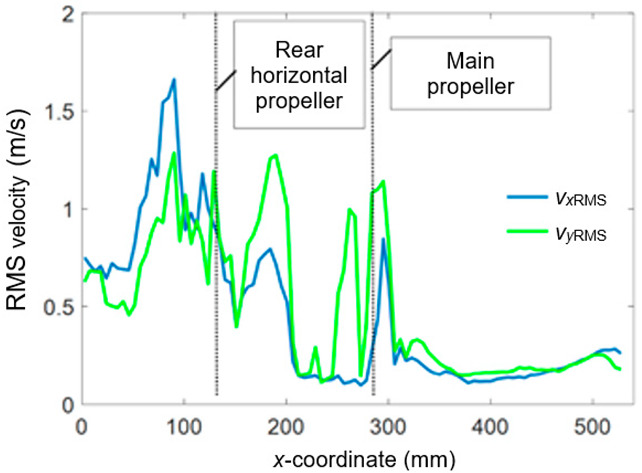
RMS velocity profile as a function of the *x*-coordinate (*v_x_*—green line, *v_y_*—blue line).

**Figure 12:**
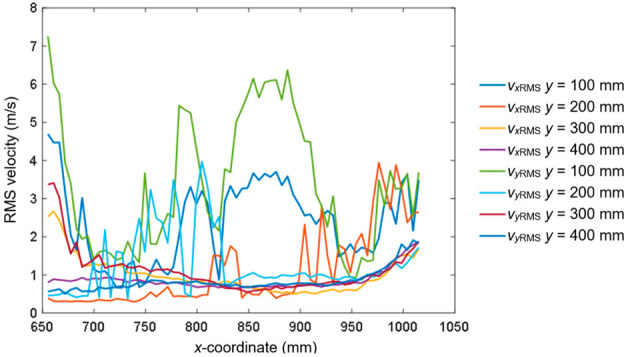
The *v*_RMS_ profiles along the *x*-coordinate for *y* = 100, 200, 300, and 400 mm.

Figure 11 presents the RMS velocity profile as a function of the *x*-coordinate for *v* = 12 m/s, α = 0°, and *y* = 720 mm. Figure 12 shows the *v*_RMS_ profiles along the *x*-coordinate for the selected values of the *y*-coordinate.

The experimental data acquisition and evaluation of the particle image recordings were performed according to best practices (i.e., the selection of tracer particles, imaging optics, optimal time between laser pulses, and seeding density listed in Reference [7]). The laser plane lens was located outside the measurement space above the research object. Such a location caused the area to be obscured (covered), e.g., under the engine or (partially) by the propeller itself. At the stage of planning the experiment, alternative locations of the laser were considered. Locating the laser behind the research object would also lead to certain areas being obscured (in this case before the engine and the propeller). In addition, the laser plane lens would be placed inside the measurement space where the air flows along with the tracer particles. After analyzing possible variants of the laser plane lens settings, the variant described in this paper was chosen.

The analysis of the images obtained during the tests was carried out using the DynamicStudio 6.11 software from Dantec Dynamics (Skovlunde, Denmark). The vector velocity fields for a pair of particle images were generated using the adaptive correlation function, which was based on the correlation analysis [18]. The final image size, after three steps of reducing the grid, was 64 × 64 px with a 50% overlap of the image position in the grid. The obtained vector fields of the displacement in pixels were scaled using a calibration procedure using calibration photos. Therefore, it was possible to convert from pixels per second to meters per second. Post-processing was used to remove the individual vectors with values that exceeded physically obtainable speeds under the given conditions and to supplement places in the vector field where the fluid velocity was not determined. Thanks to the median and averaging filter, it was possible to conduct the data processing procedure.

## 3. Results and Analysis

The tests were carried out for the following aircraft configuration: fuselage, without pusher propeller, with the main rotor, and with 1’’ diameter horizontal propellers operating at 100% of the available power (the measurement plane passed through the propeller axes with the vertical thrust vector). The front propeller was also working during the PIV measurements. The results were visualized on a plane passing through the axes of additional multirotor aircraft propellers.

**Figure 13:**
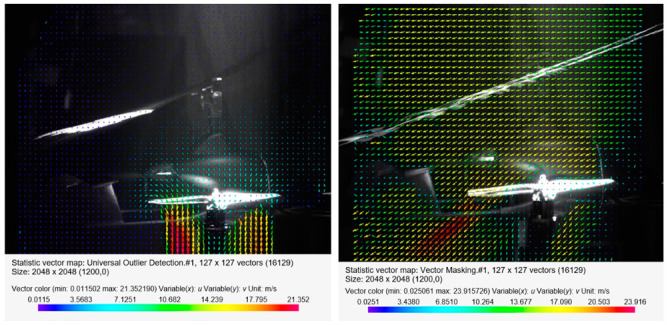
Velocity field around the horizontal propeller for the following configuration: angle of attack *α* = 0°, without airflow (on the left) and with airflow *v_wt_* = 12.5 m/s (on the right).

**Figure 14:**
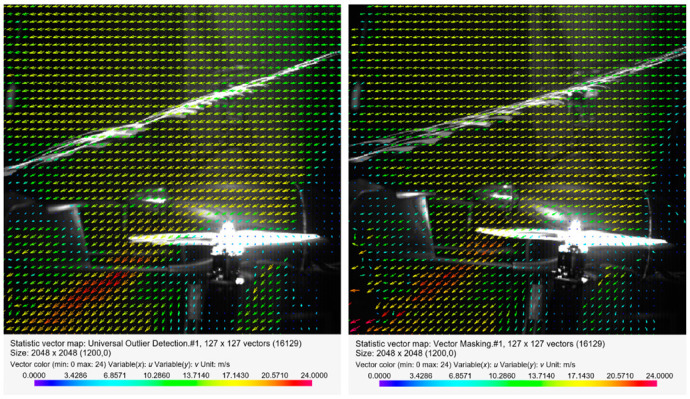
Velocity field around the horizontal propeller for the following configuration: angle of attack *α* = −4° (on the left) and angle of attack *α* = −8° (on the right), with an airflow velocity of *v_wt_* = 12.5 m/s.

**Figure 15:**
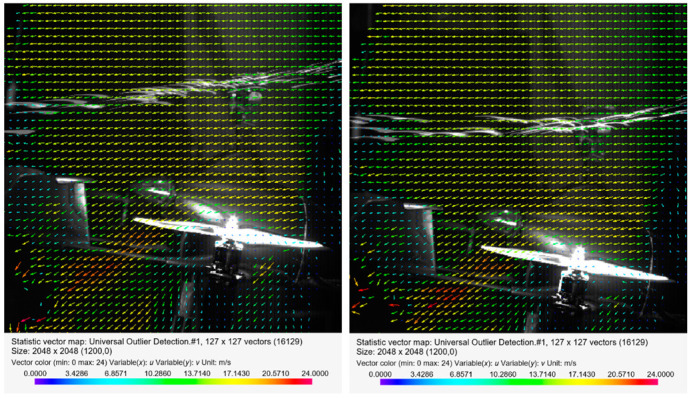
Velocity field around the horizontal propellers for the following configuration: angle of attack *α* = −12° (on the left) and angle of attack *α* = −13.5° (on the right), with an airflow velocity of *v_wt_* = 12.5 m/s.

Figure 13, Figure 14 and Figure 15 present the visualization of the aircraft flow around in the configuration described above for the angles of attack *α* equal to 0°, −4°, −8°, −12°, and −13.5°. The presented results are qualitative, while the quantitative analysis is presented in a further part of this paper.

In the first phase of the tests, the distribution of velocities around the aircraft was checked for a 0° angle of attack in the absence of airflow in the wind tunnel and with airflow at a velocity of 12.5 m/s (Figure 14). In both cases, the propellers of the additional propulsion system operated at 9240 rpm. Figure 13 shows a view of the propeller located at the rear of the fuselage. In the case of no airflow for the 0° angle of attack, asymmetry was observed in the velocity values under the tested rotor disk. Due to the local flow disturbance, among others, resulting from the presence of the fuselage, higher velocity values in the stream behind the horizontal rotor occurred in the zone closer to the stabilizers. The flow through the rotor was almost vertical. The presence of airflow at a velocity of 12.5 m/s inside the tunnel meant that the stream behind the horizontal propeller was curved in the normal direction to the airflow direction at an angle of approximately 45°. Similarly to the first case, asymmetry of the velocity value in the stream behind the horizontal propeller was observed.

The next part of the research focused on analyzing the velocity distribution for several defined values of the angle of attack in the presence of airflow at a velocity of 12.5 m/s inside the wind tunnel. This angle had a negative value, which means that the body was pitched forward, just as during the forward flight scenario without an active main rotor.

Figure 14 shows the distribution of the velocity field for the angle of attack *α* = −4° and *α* = −8° with airflow inside the wind tunnel. In the case of *α* = −4° compared to the 0° angle of attack, the maximum velocity area shifted to the left toward the stabilizers. At the end of this zone, a turbulent area was created in which the velocity vectors were not arranged in an orderly manner. An increase in the angle of attack up to *α* = −8° resulted in a disappearance of the characteristic area of maximum velocity. However, there remained a high-velocity area between the end of the propeller blade and the stabilizers of the aircraft.

Figure 15 shows a distribution of the velocity field for increased values of the angle of attack, *α* = −12° and *α* = −13.5° relative to the airflow inside the tunnel. For an angle of 12°, the characteristic area of increased velocity again appeared behind the end of the rear blade. The formation of an additional area of increased velocity in the form of a parallel zone near the engine head should be emphasized. An analysis of the velocity distribution for the angle of attack equal to −13.5° indicated an increase in the maximum velocity value behind the propeller. In the front of the rotor disk (viewed from the front of the fuselage), the velocity was close to the velocity of the airflow. This meant that the largest velocity field gradient occurred toward the stabilizers. In both considered cases, a turbulent area was formed at the end of the stream.

**Figure 16:**
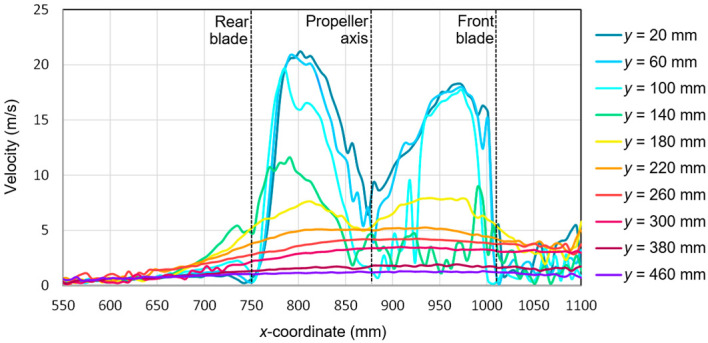
Resultant airflow velocities around the research object for the following configuration: angle of attack α = 0° and airflow in the wind tunnel *v_wt_* = 0 m/s.

**Figure 17:**
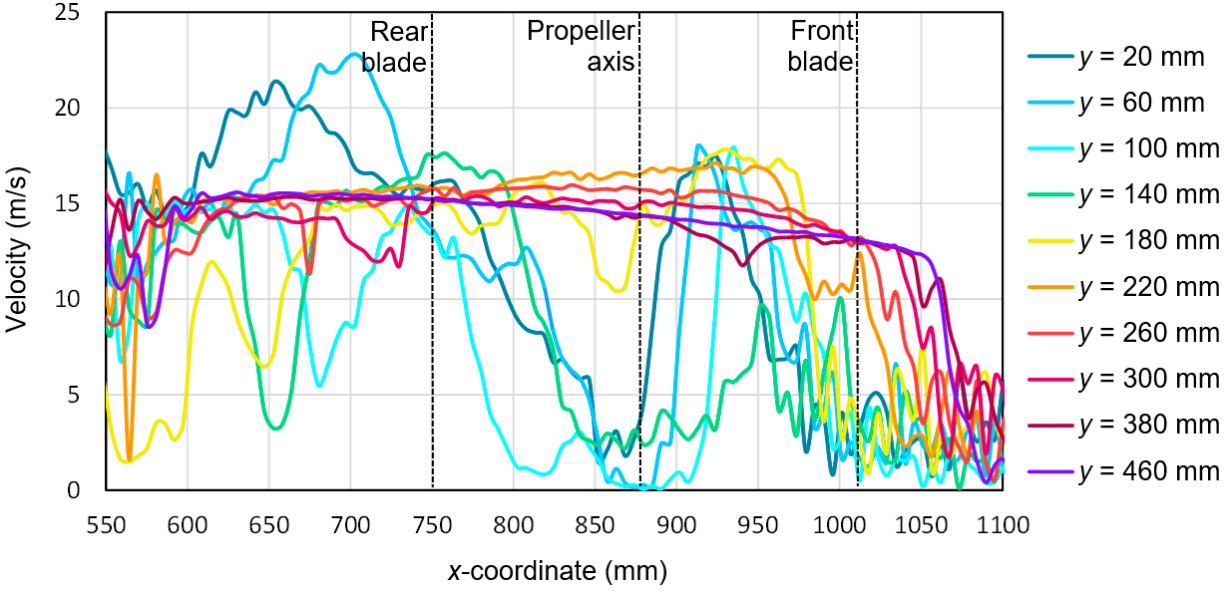
Resultant airflow velocities around the research object for the following configuration: angle of attack *α* = 0° and airflow in the wind tunnel *v_wt_* = 12.5 m/s.

**Figure 18:**
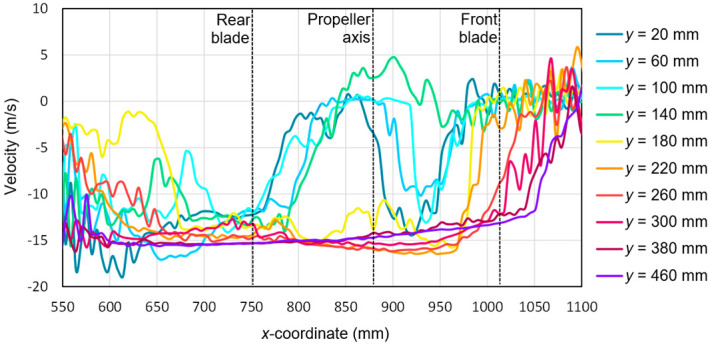
Horizontal components of the airflow velocities around the research object for the following configuration: angle of attack *α* = −4° and airflow in the wind tunnel *v_wt_* = 12.5 m/s.

**Figure 19:**
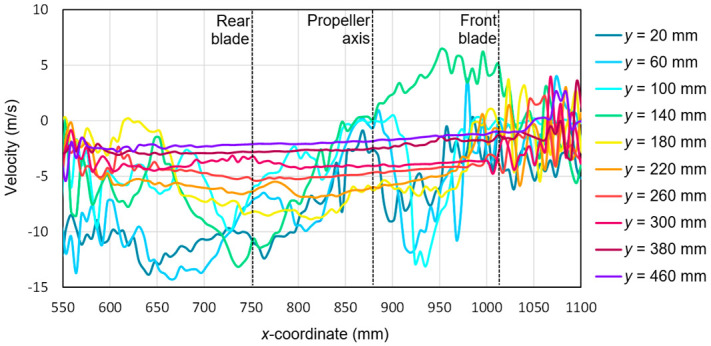
Vertical components of the airflow velocities around the research object for the following configuration: angle of attack *α* = −4° and airflow in the wind tunnel *v_wt_* = 12.5 m/s.

**Figure 20:**
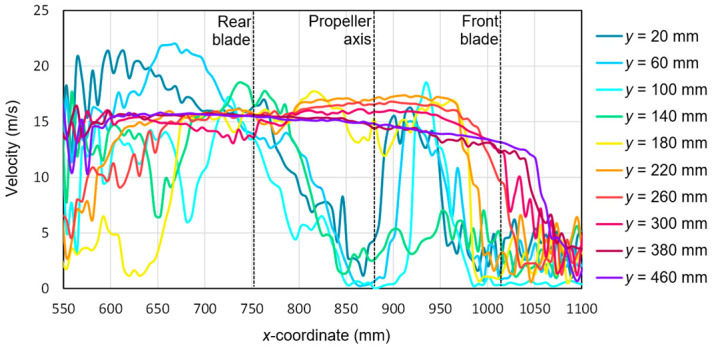
Resultant airflow velocities around the research object for the following configuration: angle of attack *α* = −4° and airflow in the wind tunnel *v_wt_* = 12.5 m/s.

**Figure 21:**
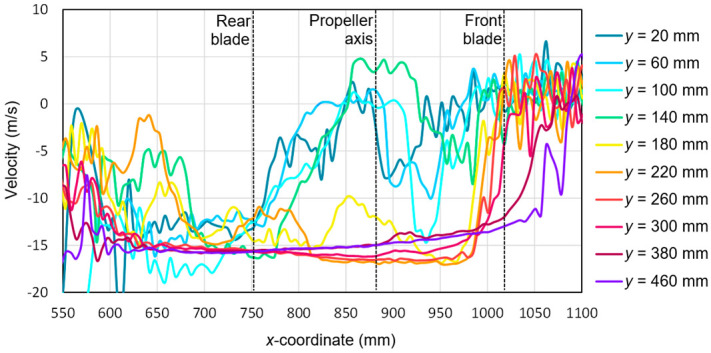
Horizontal components of the airflow velocities around the research object for the following configuration: angle of attack *α* = −12° and airflow in the wind tunnel *v_wt_* = 12.5 m/s.

**Figure 22:**
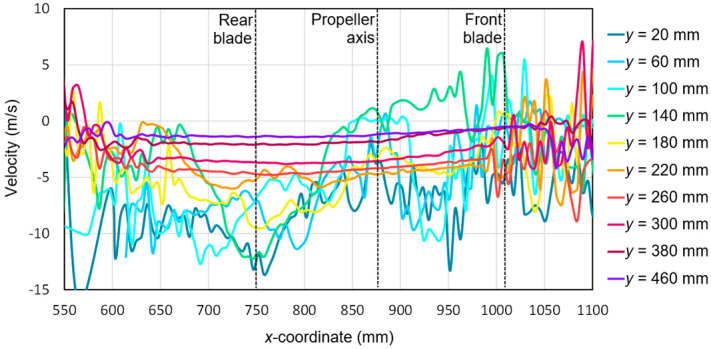
Vertical components of the airflow velocity around the research object for the following configuration: angle of attack *α* = −12° and airflow in the wind tunnel *v_wt_* = 12.5 m/s.

**Figure 23:**
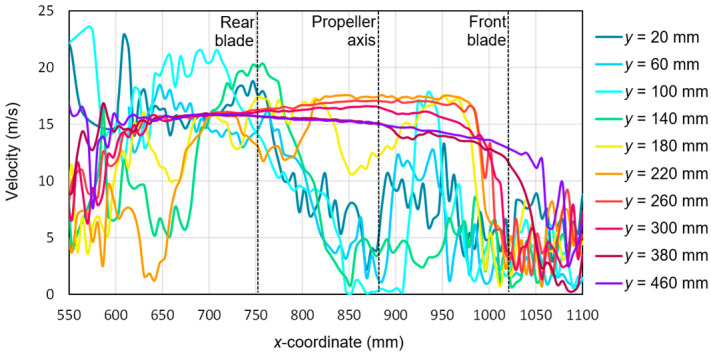
Resultant airflow velocities around the research object for the following configuration: angle of attack *α* = −12° and airflow in the wind tunnel *v_wt_* = 12.5 m/s.

Based on the tests performed using the PIV technique for the considered configuration of the test object, the airflow velocity profiles were developed as a function of the distance from the propellers. On this basis, it was possible to conduct a quantitative analysis aimed at determining the impact range of an additional multirotor propulsion system. The analysis of the velocity field around the tested object made it possible to determine the mounting location (height) of the main rotor working in autorotation relative to the fuselage and other components to minimize the impact of the additional propulsion system. Figure 16, Figure 17, Figure 18, Figure 19, Figure 20, Figure 21, Figure 22 and Figure 23 show the course of the resultant airflow velocity and its horizontal and vertical components around the considered object in the tested aircraft configuration for the selected values of the angle of attack *α* equal to 0°, –4°, and –12°. The air velocity *v* in the tunnel for the presented configurations was 0 m/s and 12.5 m/s.

When analyzing the resultant velocity for the angle of attack *α* = 0° in the absence of airflow in the tunnel, it was observed that the highest velocity value of approximately 21 m/s occurred for the coordinate *x* = 800 mm, i.e., for the center of the length of the rear blade. The highest values for the considered value of *x* were recorded for *y*, ranging from 20 to 140 mm. For the advancing blade, the maximum velocity value in the area just below its center of length was lower and amounted to 18 m/s. This confirmed the asymmetry of the velocity field distribution in the stream behind the horizontal propeller described above. For the distance *y* = 140 mm, there was a sudden change in the resultant velocity compared to, e.g., *y* = 20, 60, or 80 mm. It is worth mentioning that *y* = 140 mm was the level at which the propeller was located. The highest flow velocity value was achieved by the stream located approximately 20 mm from the plane of the propeller. For the *x*-coordinate corresponding to the rotor axis, a reduction in the resultant velocity value was observed.

The presence of the forced airflow in the tunnel with a velocity of 12.5 m/s resulted in a significant change in the recorded velocity profiles (Figure 17). In the area in front of the tested propeller (*x* from 1000 to 1100 mm) and behind it near the stabilizers (*x* from 550 to 650 mm), there was a strong flow disturbance zone, as seen by the fluctuations in the velocity values. At a sufficiently large distance from the plane of the tested propeller of the aircraft, i.e., *y* > 200 mm, the resultant velocity was approximately 15 m/s. A slightly higher value than the set air velocity inside the tunnel was due to interference with the aircraft fuselage elements and the presence of a local flow disturbance. Nevertheless, in the area of *x* ranging from 600 mm to 750 mm, two characteristic extremes exceeding 20 m/s were distinguished. They corresponded to a cutting plane at distances of 20 mm and 60 mm. This means that the area of the maximum airflow velocity shifted from the *x*-coordinate corresponding to the center of the rear blade to the coordinate corresponding to a distance of 50 to 100 mm behind its end.

The horizontal velocity component for the angle of attack *α* = −4° with the airflow inside the tunnel is shown in Figure 18. At a sufficiently large distance from the tested propeller, the absolute value of this flow velocity component was close to 15 m/s. Similarly, as in the cases described above, the difference between the given velocity of 12 m/s was due to the effect of aerodynamic interference with the rest of the aircraft parts. As in the case of a 0° angle of attack for the *x*-coordinate in the range from 550 to 650 mm and from 1000 to 1100 mm, there was a strong flow disturbance that resulted in the fluctuation of the measured component value. In the vicinity of the propeller (*x* equal to 800 to 950 mm), the horizontal velocity component values for the cutting planes located a short distance from the propeller plane (*y* = 140–160 mm) were recorded as decreasing to approximately zero. This was due to the intense propeller action, which generated a flow perpendicular to the main flow in the tunnel. The flow generated by the propeller in this region was perpendicular to the measurement plane, thus a significant loss of the particle pairs occurred. At the same time, for these cross-sections, an asymmetry of this velocity component was observed relative to the propeller center, i.e., relative to the coordinate *x* = 880 mm.

The vertical component for the angle of attack *α* = −4° with the airflow in the tunnel is shown in Figure 19. Similar to the horizontal component, at the beginning and end of the analyzed area, a characteristic flow disturbance zone was distinguished, in which the velocity values for individual components were subject to intense fluctuations. As the cutting planes moved away from the propeller plane, the velocity component value approached zero. The zero value was not reached due to an aerodynamic interference with the aircraft fuselage components. The characteristic shape had a velocity for the cutting plane at a distance of *y* = 140 mm, corresponding to the plane of the tested propeller. A change in the sign of the velocity value for this plane was observed after passing through the propeller axis, i.e., the coordinate *x* = 880 mm. Initially, a positive value of 6 m/s decreased and reaches a velocity of −13 m/s on the other side of the rotor disk. Extreme velocity values were recorded for the cross-sections *y* = 20 mm and *y* = 60 mm, which were approximately −13.5 m/s. These values occurred approximately 100 mm behind the end of the rear blade.

With both components, it was possible to determine the resultant velocity for the considered configuration (Figure 20). The velocity values obtained as a result of the calculations had a positive value for the whole considered range of *x*- and *y*-coordinates. Extreme values occurred for *y* = 20 mm and *y* = 60 mm and were equal to 22.5 m/s and 23 m/s, respectively. The lowest velocity values close to zero were recorded for the area corresponding to the propeller axis, i.e., *x* from 850 to 900 mm and for *x* equal to 1000 to 1100 mm, i.e., for the area just before the end of the front blade. Similarly for *α* = −4°, as in the case of *α* = 0°, the maximum value was reached at a distance from the propeller equal to approximately 80–120 mm. 

Figure 21 presents the horizontal components of the airflow velocities around the research object for the angle of attack *α* = −12° and the airflow velocity in the tunnel *v_wt_* = 12.5 m/s. Similarly to a smaller angle of attack in the area between the propeller and the stabilizers, there was a strong flow disturbance area in which the component of the horizontal velocity had a negative sign and locally exceeded the absolute value of 20 m/s (for *x* equal to 550 to 700 mm). The velocity value fluctuated from zero to −15 m/s. A strong flow disturbance also occurred in the area in front of the propeller (*x* equal to 1000 to 1100 mm), except that the velocity value oscillated from −5 to 5 m/s. At a greater distance from the rotor (*y* > 200 mm) the velocity stabilized and was approximately −15 m/s. The slightly higher velocity than the set airflow velocity was due to the effect of local air acceleration in the area of the aircraft fuselage. The increase in aircraft pitch (reduction of the angle of attack from −4° to −12°) did not qualitatively change the distribution of the vertical velocity component in the areas behind and in front of the propeller. In both cases, a characteristic flow disturbance zone was present. The largest significant difference occurred for the cross-section *y* = 20 mm in the immediate vicinity of the rotor disk for *x* from 700 to 950 mm. In this case, the increase in the pitch angle of the aircraft caused a decrease in the horizontal component from −15 m/s to −8 m/s, which indicated a decrease in the impact of the forced airflow on the value of the horizontal component in this zone.

In the case of the vertical velocity components (Figure 22), there were also two characteristic areas of flow disturbance occurring in front of and behind the propeller. At a greater distance from the propeller (*y* > 200 mm), the velocity stabilized for almost the entire *x*-coordinate range. The further away from the propeller, the closer to zero the velocity value became. A slightly negative value of the component (from −1 to −5 m/s) resulted from the induction of velocity due to an interference with the aircraft fuselage. Compared to the configuration without airflow in the wind tunnel for *α* = 0°, the extreme velocities in the stream behind the horizontal propeller ranged from −13 to −10 m/s. Around the propeller axis (*x* = 880 mm), the velocity component decreased and oscillated around −5 m/s. A particular trend was observed for the coordinate *y* = 140 mm, which corresponded to the propeller plane. As the *x*-coordinate increased (moving toward the front of the aircraft), the vertical component changed sign after passing through the propeller axis. The positive values were the result of the flow disturbance present on the propeller blade. The increase in the pitch angle of the aircraft resulted in an increase in the vertical component fluctuations for the cross-sections close to the plane of the rotor disk in the propeller region (*x* from 550 to 750 mm). This effect was identical to the effect observed for the horizontal component. In addition, a significant change in this velocity component was observed for the cross-section *y* = 140 mm, which corresponded to the initial plane of the rotor disk. The velocity on the side of the front blade was then closer to zero. For this region, the velocity for cross-sections in the close vicinity of the propeller also slightly decreased, i.e., for *y* = 60 mm and *y* = 100 mm.

Finally, the resulting airflow velocity was calculated for the configuration considered below (Figure 23). Comparing the obtained values for the angle of attack *α* = −4° and *α* = −12°, their greater dynamics was observed in the case of a larger pitch angle of the aircraft. Therefore, the curves had irregular shapes with numerous local extremes. This was the effect of swirling due to the flow separation at an increased angle of attack. The maximum velocity values did not change significantly and ranged from 22.5 m/s to 23.5 m/s for the area in the stream behind the horizontal propeller. The largest difference was observed for the cross-section *y* = 100 mm, in which an extreme value of about 22 m/s was observed for a larger pitch angle of the aircraft. The velocity in the area of the blade tip also intensified, i.e., for *x* = 750 mm for the cross-section *y* = 20 mm. This is seen in Figure 15 in the form of an additional high-velocity area that was not clearly visible for the smaller value of the angle of attack (Figure 14). In addition to the above-mentioned fluctuations, the largest velocity differences were observed for cross-section fragments running directly under the propeller, i.e., for *x* from 750 to 1000 mm and *y* from 20 mm to 60 mm. The increase in the pitch angle of the propeller plane resulted in the most significant change in flow conditions in the area just below the rotating blades.

## 4. Discussion and Conclusions

This paper presents the results of field velocity tests around a hybrid aircraft. The PIV technique was used to record the velocity values. The configurations with airflow forced by a fan inside the wind tunnel and without airflow were analyzed. The range of impact of one of the additional horizontal propellers and the impact of the aircraft fuselage on the recorded velocity field were analyzed. In addition, how the angle of attack affected the vertical and horizontal components and the resultant air velocity around additional propellers installed on the aircraft was also investigated.

A significant impact of the airflow on the velocity distribution around the tested propeller was observed. In the absence of airflow, there were two characteristic extremes surrounded by the centers of the front and rear blades. The resultant velocity value for the front blade was lower than the value for the rear blade by approximately 3 m/s. The observed asymmetry was the result of an aerodynamic interference with individual elements of the aircraft. In the case of airflow in the zone in front of the propeller and in the stream behind it, a strong flow disturbance zone was created, which caused fluctuations of the resultant velocity value for all considered cross-sections. Importantly, the area of the highest velocity was shifted from the center of the blades toward the stream behind the horizontal propeller.

The increase in the angle of attack did not significantly affect the vertical and horizontal components. As for the 0° angle of attack, the presence of the airflow caused a strong flow disturbance zone in front of and behind the propeller. The largest differences, in the form of an intensification of the velocity value fluctuations, occurred in the case of cross-sections located close to the rotor disk (*y* equal to 20 mm and 60 mm) in the stream behind the horizontal propeller (*x* equal to 550 to 750 mm) and in the area directly below the rotor disk (*x* equal to 750 to 900 mm). It should be mentioned that the *y* position of the disk changed with the angle of attack.

The set flow velocity in the tunnel (12 m/s) significantly differed from the velocity recorded in undisturbed cross-sections (15 m/s). This was the effect of aerodynamic interference, which should be taken into account when analyzing the velocity and pressure field around the research object.

The impact of the propeller ahead of its disk was definitely weaker than behind it. At a distance of 40 mm behind the propeller, the air reached a velocity of approximately 19.5 m/s, while at the same distance before it, the velocity was 7.5 m/s. When the velocity behind the propeller at a distance of 80 mm increased to 21 m/s, then the velocity in front of the propeller decreased to 5 m/s. At a distance of 120 mm behind the propeller, the maximum velocity value was also approximately 21 m/s, while at the same distance in front of the propeller, the maximum velocity value was 4 m/s. Up to this distance, the velocity behind the propeller increased with the distance, whereas in front of the propeller, this relationship was the opposite. A gradual decrease in velocity was observed as the distance increased. This was an obvious tendency, but without research, it is impossible to quantify the range of this impact. At a height of *y* = 380 mm, the maximum resultant velocity was less than 2 m/s. This was the height at which the main rotor was mounted (according to the additional reference system). This situation occurred without the main airflow in the wind tunnel. In the presence of airflow in the tunnel, the impact was further reduced before the propeller. This was a positive effect because it confirmed the validity of the concept of the main drive unit supported by additional propellers operating in the horizontal plane. The main rotor operation was not disturbed by additional propellers.

## Figures and Tables

**Table 1:** Technical parameters of the tested aircraft.

Parameter	Value
Rotor diameter	1100 mm
Body dimensions with stabilizers (l × h × w)	633 × 178 × 294 mm
Diameter of the three-blade push propeller	228.6 mm
Diameter of horizontal propellers	254.0 mm
Push propeller engine	ROXXY BL-Outrunner 3548/05 (Robbe, Inzersdorf im Kremstal, Austria)
Horizontal propeller engine	T-MOTOR MT2814 770 KV (T-Motor, Nanchang, Jiangxi, China)
